# From 2-acetyl-indan-1,3-dione to 2*H*-1,5-benzodiazepines and their versatile applied features

**DOI:** 10.1063/4.0000856

**Published:** 2025-10-27

**Authors:** Gordana Pavlović, Anife Ahmedova, Marin Marinov

**Affiliations:** 1 University of Zagreb Faculty of Textile Technology, Zagreb, Grad Zagreb, Croatia; 2 Faculty of Chemistry and Pharmacy, Sofia University, Sofia, Sofia, Bulgaria; 3 Agricultural University – Plovdiv, Department of Chemistry and Phytopharmacy, Faculty of Plant Protection and Agroecology, Plovdiv, Plovdiv, Bulgaria

## Abstract

Benzodiazepines (seven-membered heterocyclic compounds having two nitrogen atoms at different positions; BZDs) are ruling scaffolds in the area of the pharmaceutical industry for preparing various drugs having biological activity on central nervous system (antidepressant, anticonvulsant, muscle relaxant, anxiolytic, antiepileptic, hypnotic, and sedative functions). The most common synthesis of BZDs involves the condensation of *o*-phenylenediamine (OPD) with various carbonyl compounds (such as 1,3-dicarbonyls) in the presence of various catalysts or catalyst- free systems.

The reaction of 2-acetyl-1,3-indanedione (Scheme 1.; I) with various benzaldehydes (Scheme 1.; II) results in the cinnamoyl derivatives formation (Scheme 1; III) which upon condensation with 1,2-diaminobenzene (OPD) form the series of 2*H*-1,5-benzodiazepines.

(II: **1**, benzaldehyde; **2**, *p*-fluoro-benzaldehyde; **3**, *p*-chloro-benzaldehyde; **4**, *p-*methyl-benzaldehyde; **5**, *p*-cyano- benzaldehyde; **6**, *p*-methoxy-benzaldehyde; **7**, *p*-dimethylamino-benzaldehyde)

**Scheme 1.** Reaction scheme from 2-acetyl-1,3-indanedione (I) to cinnamoyl derivatives (III) The solid-state structural features of the aforementioned precursors as well as of the 2*H*-1,5-benzodiazepine derivative (Scheme 1., Fig.1.) prepared by us, will be discussed.

